# Violence, mental health and nutritional status in pregnant women: the Araraquara Cohort Study

**DOI:** 10.1017/S1368980024002295

**Published:** 2024-11-26

**Authors:** Leonardo Domingos Biagio, Delanjathan Devakumar, Paula Louro da Silva, Rossana Verónica Mendoza López, Perla Pizzi Argentato, Liania Alves Luzia, Patrícia Helen Carvalho Rondó

**Affiliations:** 1 Nutrition Department, School of Public Health, University of São Paulo (USP), São Paulo, Brazil; 2 Institute for Global Health, University College London (UCL), London, UK; 3 São Paulo State Cancer Institute (ICESP), São Paulo, Brazil

**Keywords:** Nutritional status, Pregnant women, Violence, Mental health

## Abstract

**Objective:**

To investigate the relationship between violence and the nutritional status of pregnant women, and whether mental health could be a mediator in this relationship.

**Design:**

Cross-sectional study. Violence and mental health status were investigated using the following questionnaires: WHO-Violence Against Women (WHO-VAW), Abuse Assessment Screen (AAS), Patient Health Questionnaire (PHQ-9) and General Health Questionnaire (GHQ). Demographic, socio-economic, obstetric and lifestyle factors (smoking/alcohol consumption) were also investigated. The nutritional status of the women was assessed by the BMI.

**Setting:**

Data were collected from February 2021 to August 2022 in Araraquara city, Brazil.

**Participants:**

Four hundred pregnant women recruited at thirty-four health units and the municipal maternity hospital.

**Results:**

Experience of violence was reported by 52·2 % of the women, and psychological violence in the last 12 months was the most prevalent type of domestic violence (19·5 %). Approximately 43 % of the women showed mental health changes and 59·7 % had a risk of major depression. Women with mental health changes had an increased risk (OR = 2·34) of obesity. Psychological violence in the last 12 months was associated with obesity (*P* = 0·01) when mediated by mental health changes. The mediation effect was significant (*β* = 0·708; 95 % bias-corrected and accelerated (BCa) CI = 0·004, 1·460), with mental health changes mediating 46·1 % of the relationship between psychological violence and obesity.

**Conclusions:**

The relationship between psychological violence and obesity during pregnancy was mediated by changes in mental health. This original study shows that nutritional status is not limited to biological factors and highlights the importance of social, mental and psychological factors.

According to the WHO, 30 % of women worldwide suffered physical and sexual violence between 2000 and 2018^(1)^. Pregnancy can be a particularly vulnerable period for domestic violence (DV) because of the physical and emotional changes that occur during this period. Moreover, social and economic status may increase this risk in minority groups. A review conducted in 2021 that involved seven systematic reviews and five meta-analyses reported a prevalence of psychological and physical DV during pregnancy ranging from 1·8 to 67·4 % and from 1·6 to 78 %, respectively. The reviews involved high, but mainly low- and middle-income countries and showed a higher risk of violence in the latter countries due to social factors^(2)^. In a meta-analysis involving data from Brazil, James *et al.* ^(3)^ found an overall prevalence of violence of 63·40 % (40·40 % of psychological DV, 12·35 % of physical DV and 4·55 % of sexual DV) in pregnant women.

Pregnant women exposed to DV are more susceptible to mental health changes such as depression, anxiety, emotional distress, suicidal ideation and lack of self-efficacy and self-esteem^(4,5)^. A recent systematic review and meta-analysis^(6)^ assessed the prevalence of DV and the magnitude of the association between violence and mental health outcomes in 250·599 women who were primarily from high-income countries. Lifetime psychological violence was reported by 32·8 % of the women, while physical and sexual violence was reported by 18·3 % and 9·6 %, respectively. Perinatal women were more likely to have experienced lifetime physical DV (28·8 %) than the other women investigated (women in the community and help-seeking women). The meta-analysis suggested an association between DV and an increased risk of all mental health outcomes, including depression, posttraumatic stress disorder and suicidality.

Violence against women has a negative impact not only on mental health but also on physical health, considering that stress can increase the concentrations of glucocorticoids and insulin influencing eating behaviour by either increasing or decreasing food intake and affecting metabolism^(7–9)^. Therefore, some mental health changes, particularly stress, have a bidirectional effect on food intake, and women may thus be either under- or overweight.

Chronic stress has been found to cause weight loss by increasing the metabolic rate and energy expenditure^(7,8)^. Conversely, long-term chronic stress can trigger prolonged hyperactivation of the hypothalamus–pituitary–adrenal axis, with further increases in abdominal fat retention and TAG concentrations, that lead to overweight and obesity^(7,10,11)^.

In a cohort of 206 pregnant women, Farias *et al.* ^(12)^ observed that persistent depressive symptoms resulted in a 2·40 times higher risk of insufficient gestational weight gain. However, the authors found no significant association of generalised anxiety disorder or suicide risk with weight gain. In a Thai study^(13)^, pregnant women with high scores on depression and anxiety scales had a higher BMI during pregnancy. The results are similar to the findings of two meta-analyses in which severe symptoms of depression were associated with excessive gestational weight gain^(14,15)^.

Studies on non-pregnant women suggest that DV can influence their nutritional status in different ways, but the results are limited and inconsistent^(16–19)^. To our knowledge, there is only one large epidemiological study that has evaluated the association between prenatal depressive symptoms, maternal nutrition, DV and social support among pregnant women in rural Ethiopia. The authors observed that several factors were associated with depression, including DV, mid-upper arm circumference less than 23 cm and anaemia^(20)^. Furthermore, maternal mental health changes, either directly or as a result of DV, may compromise their offspring earlier in utero, predisposing to low birth weight or premature labour^(21,22)^, and even to later malnutrition^(23,24)^.

Apparently, the number of studies evaluating the impact of DV and mental health changes on maternal nutritional status is much smaller than the number of studies evaluating the impact of DV and mental health changes on adverse outcomes in offspring.

Therefore, this study investigated the relationship between violence and the nutritional status of pregnant women, and whether mental health could be a mediator in this relationship.

## Materials and methods

This cross-sectional study investigated violence and mental health in 400 pregnant women. The study is part of a large epidemiological prospective cohort – the Araraquara Cohort Study – that assessed the relationship between maternal and offspring adiposity during the fetal, neonatal and infant periods. Pregnant women with a gestational age ≤26 weeks were included in this study.

Data were collected from February 2021 to August 2022. The women were selected by trained interviewers at the thirty-four health units and at the municipal maternity hospital of Araraquara city, São Paulo, Brazil. The participants answered a questionnaire previously used in pregnant women^(25)^, which contained data on demographic, socio-economic, obstetric and lifestyle factors (smoking/alcohol consumption). The women attended the Serviço Especial de Saúde de Araraquara (SESA) for assessment of their nutritional status and their gestational age by ultrasonography. They were also asked about violence and mental health using standardised questionnaires that were applied online.

Before the start of any conversation about violence, the interviewers were instructed to ask whether the participant felt safe to speak and she could answer simply with ‘yes’ or ‘no’. If she did not feel safe, a better time for the interview was suggested. In addition, the interviewers asked the participant if she was alone, ensuring that the abuser was not in the same room with her. The WHO ethical and safety recommendations for research on violence against women were followed (WHO, 2001).

### Questionnaires for assessing violence

#### WHO-Violence Against Women^ (26)^

The instrument consists of thirteen items that evaluate marital violence (psychological, physical and sexual) in the last 12 months. This questionnaire has been used in several international and national studies, including those involving Brazilian pregnant women^(27,28)^.

#### Abuse Assessment Screen^ (29)^

The Abuse Assessment Screen (AAS) is a specific instrument used to assess violence during pregnancy and has already been applied to Brazilian pregnant women^(29)^. The instrument contains five questions designed to identify the frequency and severity of events, the locations of bodily harm suffered over a given period and the profile of the perpetrator. The questions address lifetime experiences of abuse, physical violence in the past year, physical violence during pregnancy, sexual abuse in the past 12 months and fear of a current partner or someone close.

### Questionnaires for assessing mental health

#### General Health Questionnaire^ (30)^

The General Health Questionnaire (GHQ) measures psychological well-being in the adult population, including pregnant women^(25)^. The questionnaire addresses common mental health problems/domains of depression, anxiety, somatic symptoms and social withdrawal. It is simple to administer, easy to complete and score, and widely used in many studies^(31)^. The version used consisted of twelve questions. The GHQ score was classified as low (0–3) and high (≥4)^(30)^.

#### Patient Health Questionnaire^ (32)^

The Patient Health Questionnaire (PHQ-9) aims to assess the risk of major depression and has been validated for the Brazilian population and applied to pregnant women^(33,34)^. It consists of nine symptoms, and the frequency of each symptom is rated over the past 2 weeks on a Likert scale from 0 to 3. The total score ranges from 0 to 27, and a score ≥10 is defined as the presence of depression.

### Assessment of nutritional status

The nutritional status of the pregnant women was assessed based on BMI at gestational age ≤26 weeks. Weight was evaluated by electrical bioimpedance using the Inbody 370 equipment (Biospace®). Height was measured with a Seca 206 stadiometer (Seca®). The Atalah reference curve was used to classify the pregnant women according to their BMI^(35)^.

### Statistical analysis

Descriptive analysis was performed to obtain the frequency and percentage of the independent, dependent and confounding variables. The Shapiro–Wilk test was applied to analyse the normality of the data. The *χ* ^2^ test and Fisher’s exact test were used to evaluate the associations among the variables. Multivariate logistic regression models were built to estimate the relative risk ratios between violence and the nutritional status of the pregnant women. The models were adjusted for the following confounding variables: age, parity and gestational age. The results were reported as OR and 95 % CI, and a *P*-value <0·05 was considered statistically significant. To assess the effect of mental health as a mediator in the relationship between violence (X) and nutritional status (Y), we used the PROCESS macro for SPSS. This tool is widely employed to estimate direct and indirect effects in single and multiple mediator (parallel and serial) models. In this study, the mediation process involved only one mediating variable (mental health changes) and was therefore simple mediation – a type of mediation that occurs when one variable (*M*) mediates the effect of *X* on *Y* ^(36–38)^. Statistical analysis was performed using the SPSS® software (version 20).

## Results

A total of 471 pregnant women were recruited for the study. Seventy-one (15·1 %) women refused to participate due to the following reasons: lack of interest and lack of time. Therefore, 400 women completed the study.

Table 1 shows the demographic, socio-economic, obstetric, nutritional, and lifestyle characteristics, violence at any point in their lifetime, DV in the last 12 months, and mental health of the pregnant women studied. Most pregnant women (87 %) were 20–30 years of age and were non-White (52 %), were married or had a common-law union (87·5 %), had ≤12 years of schooling (81 %) and had a per capita income of 0·5–1 Brazilian minimum wage (56·3 %). Less than half of the women had an intimate partner as head of the household (40·5 %), and most of them did not consume alcohol, did not smoke and did not use illicit drugs during pregnancy. Almost 43 % of the women were primiparous and 78·7 % were at 14–26 weeks of gestation. The majority of pregnant women had a BMI above the adequate range (56·6 %), with similar percentages of overweight and obesity, while a minority were under weight (12·8 %).

**Table 1.** Demographic, socio-economic, obstetric, nutritional and lifestyle characteristics, violence at any point in their lifetime, DV in the last 12 months, and mental health of the pregnant women

Characteristics	*n*	%
Age (years)		
<20	30	7·5
20–30	348	87
>30	22	5·5
Race		
White	192	48
Non-White	208	52
Marital status		
Married/common-law union	350	87·5
Single/divorced/widow	50	12·5
Education (years of schooling)		
˂12	324	81
≥12	76	19
Per capita income (BMW)^*^		
<0·5	148	37
0·5–1	225	56·2
>1	27	6·8
*Head of household*		
Intimate partner	162	40·5
Pregnant woman	73	18·3
Intimate partner and pregnant woman	88	22
Other	77	19·2
Alcohol consumption		
Yes	53	13·2
No	347	86·8
Smoking		
Yes	26	6·5
No	374	93·5
Use of illicit drugs		
Yes	8	2
No	392	98
Parity		
0	171	42·7
1–2	190	47.5
≥3	39	9.8
Gestational age (weeks)		
≤13	85	21·3
14–26	315	78·7
BMI (kg/m^2^)		
Undernutrition	51	12·8
Normal	123	30·8
Overweight	113	28·2
Obesity	113	28·2
AAS (violence at any point in their lifetime)	209	52·2
WHO-VAW (DV in the last 12 months)		
Psychological	78	19·5
Physical	30	7·5
Sexual	11	2·7
GHQ		
0–3	229	57·3
≥4	171	42·7
PHQ		
0–9	161	40·3
≥10	239	59·7

DV, domestic violence; BMW, Brazilian minimum wage; AAS, Abuse Assessment Screen; WHO-VAW, WHO-Violence Against Women; GHQ, General Health Questionnaire; PHQ, Patient Health Questionnaire.

* One BMW = US$ 267.00.

Experience of violence at any point in their lifetime was reported by 52·2 % of the pregnant women. Psychological violence was the most prevalent type of DV in the last 12 months (19·5 %) followed by physical (7·5 %) and sexual violence (2·7 %). The GHQ scores revealed mental health changes in 42·7 % of the pregnant women and 59·7 % had a risk of major depression based on PHQ scores.

Table 2 shows the associations of the nutritional status of pregnant women with demographic, socio-economic, obstetric, and lifestyle characteristics, DV at any point in their lifetime, type of violence in the last 12 months, and mental health changes. There were statistically significant associations between the current nutritional status of the pregnant women and mental health assessed by the GHQ (*P* = 0·021), age (*P* = 0·001) and parity (*P* ≤ 0·001).

**Table 2.** Associations of the nutritional status of pregnant women with demographic, socio-economic, obstetric and lifestyle characteristics, DV at any point in their lifetime, type of violence in the last 12 months, and mental health changes

Variable	Undernutrition		Normal		Overweight		Obesity		*P*-value^*^
	*n*	%	*n*	%	*n*	%	*n*	%	
Age (years)									
<20	9	17·6 %	8	6·5 %	6	5·3 %	1	0,9 %	<0·001
20–30	34	66·7 %	73	59·3 %	64	56·6 %	59	52·2 %	
>30	8	15·7 %	42	34·2 %	43	38·1 %	53	46·9 %	
Per capita income (BMW)^***^									0·265
<0·5	12	23·6 %	45	36·6 %	53	46·9 %	38	33·6 %	
0·5–1	35	68·6 %	70	56·9 %	50	44·2 %	70	62·0 %	
>1	4	7·8 %	8	6·5 %	10	8·9 %	5	4·4 %	
Race									
White	26	51 %	62	50·4 %	51	45·1 %	53	46·9 %	0·827
Non-White^†^	25	49 %	61	49·6 %	62	54·9 %	60	53·1 %	
Marital status									
Married/common-law union	43	84·3 %	107	87 %	99	87·6 %	101	89·4 %	0·833
Single/divorced/widow	8	15·7 %	16	13 %	14	12·4 %	12	10·6 %	
*Head of household*									
Intimate partner	18	35·3 %	48	39 %	48	42·5 %	48	42·5 %	0·354
Pregnant woman	11	21·6 %	24	19·5 %	22	19·5 %	16	14·1 %	
Intimate partner and pregnant woman	6	11·8 %	29	23·6 %	24	21·2 %	29	25·7 %	
Other	16	31·3 %	22	17·9 %	19	16·8 %	20	17·7 %	
Education (years of schooling)									
<12	39	76·5 %	99	80·5 %	94	83·2 %	92	81·4 %	0·786
≥12	12	23·5 %	24	19·5 %	19	16·8 %	21	18·6 %	
Smoking									
Yes	45	88·2 %	119	96·7 %	107	92·9 %	105	92·9 %	0·205
No	6	11·8 %	4	3·3 %	8	7·1 %	8	7·1 %	
Use of illicit drugs									
Yes	49	96·1 %	121	98·4 %	109	96·5 %	113	100 %	0·125^**^
No	2	3·9 %	2	1·6 %	4	3·5 %	0	0 %	
Alcohol consumption									
Yes	40	78·4 %	102	82·9 %	84	74·3 %	90	79·6 %	0·447
No	11	21·6 %	21	17·1 %	29	25·7 %	23	20·4 %	
Gestational age (weeks)									
≤13	10	19·6 %	31	25·2 %	24	21·2 %	20	17·7 %	0·556
14–26	41	80·4 %	92	74·8 %	89	78·8 %	93	82·3 %	
Parity									
0	33	64·7 %	58	47·2 %	37	32·8 %	43	38·1 %	<0·001
1–2	14	27·5 %	55	44·7 %	64	56·6 %	57	50·4 %	
≥3	4	7·8 %	10	8·1 %	12	10·6 %	13	11·5 %	
GHQ									
0–3	30	58·8 %	83	67·5 %	62	54·9 %	54	47·8 %	0·021
≥4	21	41·2 %	40	32·5 %	51	45·1 %	59	52·2 %	
PHQ									
0–9	17	33·3 %	61	49·6 %	42	37·2 %	41	46·3 %	0·083
≥10	34	66·7 %	62	50·4 %	71	62·8 %	72	63·7 %	
WHO-VAW-psychological violence									
No	39	76·5 %	97	78·9 %	90	79·6 %	96	85 %	0·528
Yes	12	23·5 %	26	21·1 %	23	20·4 %	17	15 %	
WHO-VAW-physical violence									
No	45	88·2 %	114	92·7 %	106	93·8 %	105	92·9 %	0·648
Yes	6	11·8 %	9	7·3 %	7	6·2 %	8	7·1 %	
WHO-VAW-sexual violence									
No	49	96·1 %	118	95·9 %	113	100 %	109	96·5 %	0·107^**^
Yes	2	3·9 %	5	4·1 %	0	0 %	4	3·5 %	
AAS (violence at any point in their lifetime)									
No	24	47·1 %	59	48 %	53	46·9 %	55	48·7 %	0·994
Yes	27	52·9 %	64	52 %	60	53·1 %	58	51·3 %	

DV, domestic violence; BMW, Brazilian minimum wage; GHQ, General Health Questionnaire; PHQ, Patient Health Questionnaire; WHO-VAW, WHO-Violence Against Women; AAS, Abuse Assessment Screen.

* *χ* ^2^ test.

** Fisher’s exact test.

*** One BMW = US$ 267.00.

† Non-White = Black, Mixed race, Yellow and Indigenous people.

Table 3 shows the results obtained with four multivariate regression models that assessed the associations of the nutritional status category of pregnant women with mental health and DV in the last 12 months, controlling for the confounders age, parity and gestational age. Mental health changes assessed by the GHQ were associated with an increased risk of the pregnant woman being obese (OR = 2·34) compared with women with a normal BMI (*P* = 0·002). Similarly, women with altered PHQ scores were more likely to be obese (OR = 1·81). Psychological and physical violence in the last 12 months were not associated with any of the nutritional status categories. As expected, the confounder age <20 years was positively associated with the nutritional status of the pregnant women (undernutrition) in all models.

**Table 3.** Associations of nutritional status category of the pregnant women with mental health and DV in the last 12 months

Variable	Undernutrition			Overweight			Obesity		
Model 1	OR	95 % CI	*P*	OR	95 % CI	*P*	OR	95 % CI	*P*
GHQ									
≥4	1·50	0·748, 3·022	0·253	1·61	0·936, 2·767	0·085	2·34	1·359, 4·051	0·002
0–3	Ref.			Ref.			Ref.		
Age (years)									
>30	2·12	0·858, 5·287	0·103	0·94	0·530, 1·690	0·851	0·58	0·327, 1·041	0·068
<20	4·89	1·318, 18·189	0·018	1·09	0·327, 3·661	0·884	0·10	0·012, 0·929	0·043
20–30	Ref.			Ref.			Ref.		
Parity									
0	0·56	0·150, 2·133	0·400	1·03	0·406, 2·614	0·950	0·98	0·388, 2·511	0·979
≥3	0·97	0·261, 3·634	0·968	0·59	0·220, 1·599	0·302	0·97	0·366, 2·612	0·964
1–2	Ref.			Ref.			Ref.		
Gestational age (weeks)									
0–13	1·36	0·600, 3·121	0·455	1·25	0·677, 2·338	0·468	1·53	0·801, 2·946	0·197
14–26	Ref.			Ref.			Ref.		
**Model 2**									
PHQ									
>9	1·78	0·887, 3·599	0·105	1·63	0·959, 2·773	0·071	1·81	1·062, 3·103	0·029
0–9	Ref.			Ref.			Ref.		
Age (years)									
>30	2·07	0·832, 5·148	0·117	0·94	0·527, 1·684	0·842	0·61	0·343, 1·086	0·093
<20	4·52	1·206, 16·97	0·025	1·03	0·307, 3·470	0·960	0·10	0·012, 0·885	0·038
20–30	Ref.			Ref.			Ref.		
Parity									
0	0·57	0·152, 2·180	0·417	1·04	0·412, 2·654	0·926	0·985	0·390, 2·490	0·974
1–2	0·96	0·260, 3·615	0·962	0·578	0·215, 1·55	0·278	0·886	0·335, 2·342	0·807
≥3	Ref.			Ref.			Ref.		
Gestational age (weeks)									
0–13	1·29	0·568, 2·69	0·536	1·21	0·654, 2·265	0·535	1·46	0·767, 2·800	0·247
14–26	Ref.			Ref.			Ref.		
**Model 3**									
WHO-VAW-psychological violence									
Yes	0·77	0·347, 1·747	0·544	1·14	0·602, 2·186	0·676	1·58	0·798, 3·160	0·187
No	Ref.			Ref.			Ref.		
Age (years)									
>30	2·22	0·899, 5·523	0·084	1·03	0·581, 1·83	0·913	0·70	0·399, 1·238	0·222
<20	5·081	1·369, 18·848	0·015	1·11	0·332, 3·710	0·865	0·11	0·013, 0·969	0·047
20–30	Ref.			Ref.			Ref.		
Parity									
0	0·55	0·146, 2·089	0·382	0·99	0·392, 2·514	0·987	0·90	0·357, 2·270	0·824
1–2	0·92	0·246, 3·465	0·906	0·52	0·195, 1·413	0·202	0·74	0·281, 1·962	0·548
≥3	Ref.			Ref.			Ref.		
Gestational age (weeks)									
0–13	1·38	0·608, 3·155	0·439	1·26	0·680, 2·338	0·461	0·74	0·795, 2·882	0·207
14–26	Ref.			Ref.			Ref.		
**Model 4**									
WHO-VAW-physical violence									
Yes	1·60	0·524, 4·907	0·408	0·82	0·291, 2·309	0·707	1·04	0·383, 2·836	0·936
No	Ref.			Ref.			Ref.		
Age (years)									
>30	2·22	0·897, 5·493	0·085	1·03	0·581, 1·834	0·914	0·67	0·383, 1·184	0·170
<20	4·91	1·32, 18·246	0·017	1·12	0·337, 3·765	0·847	0·11	0·013, 0·977	0·048
20–30	Ref.			Ref.			Ref.		
Parity									
0	0·53	0·141, 2·022	0·355	1·00	0·399, 2·549	0·986	0·93	0·373, 2·351	0·889
1–2	0·88	0·237, 3·278	0·851	0·53	0·201, 1·436	0·215	0·80	0·308, 2·113	0·662
≥3	Ref.			Ref.			Ref.		
Gestational age (weeks)									
0–13	1·353	0·594, 3·082	0·472	1·26	0·683, 2·347	0·454	1·52	0·801, 2·894	0·200
14–26	Ref.			Ref.			Ref.		

DV, domestic violence; GHQ, General Health Questionnaire; PHQ, Patient Health Questionnaire; WHO-VAW, WHO-Violence Against Women.

Multivariate regression models. Confounding factors included in the models: age, parity and gestational age.

**Figure 1.**
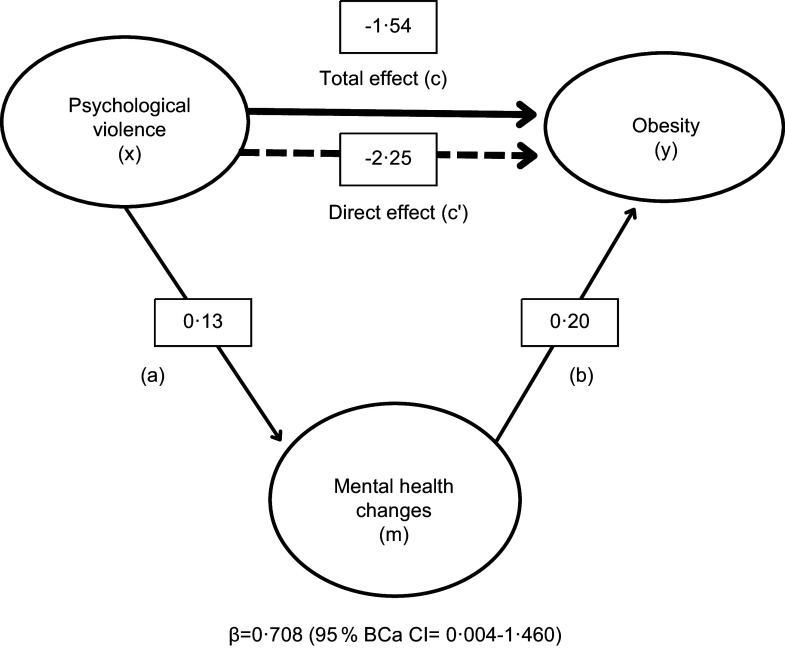
Model of psychological violence as a predictor of obesity, mediated by mental health changes. The bias-corrected and accelerated CI (BCa CI) was estimated using the bootstrapping technique (5000, re-sampling).

Table 4 shows the results obtained with multivariate regression models that assessed the associations of the nutritional status category of pregnant women with DV in the last 12 months, mediated by mental health and controlling for the confounders age, parity and gestational age. In model 5, psychological violence in the last 12 months was associated with obesity (*P* = 0·01). We sought to investigate the extent to which mental health changes mediated the relationship between psychological violence and obesity. The mediation effect (indirect effect) was significant (*β* = 0·708; 95 % bias-corrected and accelerated (BCa) CI = 0·004, 1·460). Obese pregnant women had a 2·82 times higher chance of having mental health changes than women with a normal BMI (*P* ≤ 0·001). As shown in Fig. 1, mental health changes mediated 46·1 % of the relationship between psychological violence and obesity. According to model 6, physical violence was not associated with any of the nutritional status categories. There was a 2·36 times higher chance of a pregnant woman with obesity having mental health changes compared with women with a normal BMI (*P* = 0·002).

**Table 4.** Associations of nutritional status category of the pregnant women with DV in the last 12 months, mediated by mental health

Variable	Undernutrition			Overweight			Obesity		
Model 5	OR	95 % CI	*P*	OR	95 % CI	*P*	OR	95 % CI	*P*
WHO-VAW-psychological violence									
Yes	0·90	0·380	0·825	1·45	0·730, 2·915	0·286	2·42	1·160, 5·08	<0·001
No	Ref.			Ref.			Ref.		
GHQ									
≥4	1·42	0·672, 3·002	0·358	1·75	0·980, 3·115	0·058	2·82	1·586, 5·022	<0·001
0–3	Ref.			Ref.			Ref.		
Age (years)									
>30	2·12	0·852, 5·289	0·106	0·95	0·536, 1·718	0·889	0·61	0·340, 1·093	0·096
<20	4·93	1·326, 18·385	0·017	1·06	0·319, 3·586	0·914	0·10	0·012, 0·889	0·039
20–30	Ref.			Ref.			Ref.		
Parity									
0	0·55	0·148, 2·114	0·391	1·00	0·396, 2·558	0·990	0·92	0·362, 2·374	0·873
1–2	0·97	0·259, 3·678	0·972	0·56	0·209, 1·540	0·266	0·87	0·323, 2·354	0·787
≥3	Ref.			Ref.			Ref.		
Gestational age (weeks)									
0–13	1·37	0·604, 3·148	0·445	1·25	0·673, 2·328	0·479	1·52	0·792, 2·948	0·206
14–26	Ref.			Ref.			Ref.		
**Model 6**									
WHO-VAW-physical violence									
Yes	1·39	0·438, 4·440	0·574	0·66	0·230, 1·930	0·454	0·74	0·263, 2·084	0·570
No	Ref.			Ref.			Ref.		
GHQ									
≥4	1·40	0·683, 2·859	0·360	1·64	0·944, 2·850	0·079	2·36	1·355, 4·097	0·002
0–3	Ref.			Ref.			Ref.		
Age (years)									
>30	2·11	0·849, 5·256	0·108	0·96	0·536, 1·719	0·891	0·59	0·330, 1·054	0·075
<20	4·83	1·300, 17·975	0·019	1·01	0·330, 3·709	0·870	0·10	0·012, 0·937	0·043
20–30	Ref.			Ref.			Ref.		
Parity									
0	0·54	0·144, 2·073	0·374	1·04	0·411, 2·647	0·930	0·99	0·391, 2·529	0·990
1–2	0·95	0·254, 3·563	0·940	0·59	0·222, 1·615	0·311	0·98	0·368, 2·628	0·974
≥3	Ref.			Ref.			Ref.		
Gestational age (weeks)									
0–13	1·35	0·592, 3·081	0·474	1·26	0·678, 2·36	0·463	1·53	0·799, 2·943	0·198
14–26	Ref.			Ref.			Ref.		

DV, domestic violence; WHO-VAW, WHO-Violence Against Women; GHQ, General Health Questionnaire.

Multivariate regression models, considering mental health as mediator. Confounding factors included in the models: age, parity and gestational age.

## Discussion

The objective of this study was to assess the impact of DV and mental health on the nutritional status of pregnant women. Moreover, we sought to investigate the extent to which mental health changes mediated the relationship between psychological violence and nutritional status of the women.

Psychological violence, assessed by the WHO-Violence Against Women (WHO-VAW), was more prevalent (19·5 %) than physical (7·5 %) and sexual (2·7 %) violence among the pregnant women investigated, in agreement with previous studies^(3,39,40)^. However, studies that assess DV are sensitive to methodological factors. In addition, the perception of abuse as violence is low, especially sexual violence^(41)^.

According to GHQ scores, 42·7 % of the women had mental health changes, a higher percentage than that reported in a cohort study conducted by our group, with a similar population, in which the frequency of high GHQ scores (≥4) was 30·4 % among adult pregnant women in the first trimester of gestation^(42)^. A recent study conducted in Nigeria^(43)^ detected high GHQ scores among 51·8 % of the 991 pregnant women investigated compared with 28·6 % of non-pregnant women. The PHQ-9 identified that 59·7 % of the women included in our study had depression. A Chinese study reported a lower prevalence of depression (36·12 %) among 681 pregnant women who attended various obstetrics and gynecology clinics in Changzhou city, located in the economically developed eastern part of China^(44)^.

More than half of the pregnant women (56·6 %) were overweight/obese. Nutritional status was significantly associated with GHQ and PHQ-9 scores. Multivariate regression models, controlling for the confounders age, parity and gestational age, showed that overweight/obese pregnant women had a 2·32 times higher chance of having mental changes and a 1·81 times higher chance of having major depression than women with a normal BMI. Similarly, studies conducted in different regions of the world^(13–15,45)^ found associations of excessive BMI during pregnancy with depression and anxiety. There was no association between undernutrition and GHQ or PHQ-9 scores.

Psychological and physical violence in the last 12 months were not associated with the nutritional status of pregnant women, in multivariate regression analysis controlling for the confounders age, parity and gestational age. However, in the final multivariate regression model that included mental health (GHQ scores), psychological violence was significantly associated with overweight/obesity. The inclusion of GHQ in the model showed that mental health changes acted as a mediator between psychological violence and the nutritional status of pregnant women. Obese pregnant women had a 2·82 times higher chance of having mental health changes than women with a normal BMI. Mental health changes mediated 46·1 % of the relationship between psychological violence and obesity. To our knowledge, there are no studies that assessed the relationship between DV and the nutritional status of pregnant women, considering mental health as a mediator between these two factors.

Physical violence in the last 12 months, on the other hand, was not associated with nutritional status of the pregnant women, in contrast to the findings of another Brazilian study^(18)^ involving non-pregnant women, that revealed an inverse association of physical violence with BMI. The results showed that physical intimate partner violence was negatively associated with BMI between the 25th and 85th percentiles, corresponding to 22·9 and 31·2 kg/m^2^, respectively. Adhikari *et al.* ^(19)^ did not find any association between underweight and DV among non-pregnant Nepalese women, but overweight was associated with physical violence, including severe physical violence.

We found no association between sexual violence and the nutritional status of pregnant women, probably due to the low prevalence of sexual violence (2·7 %). It was therefore not possible to include the data in the regression analysis. The experience of physical and sexual violence was a marker for increased risk of chronic undernutrition in women of reproductive age in Bangladesh^(46)^, which was more strongly accentuated among the poorest women. In a study of Nepalese women, emotional and physical violence was significantly associated with the risk of being overweight and obese, while sexual violence alone was associated with a BMI compatible with underweight^(47)^.

One limitation of our study is its cross-sectional design. Due to the COVID-19 pandemic, we were unable to follow up the women throughout pregnancy. In addition, some studies reported an increase in violence and mental health changes during the COVID-19 pandemic^(48,49)^, a fact that may have influenced the results of this study. Another limitation of the study is that we did not use a specific questionnaire to assess anxiety, only the GHQ, that measures mental health changes in general. A strength of the study is its originality in evaluating the relationship between DV, mental health changes and nutritional status in pregnant women since the studies available so far only involve non-pregnant women. Furthermore, there are no studies assessing the role of mental changes as a mediator in the relationship between violence and the nutritional status of pregnant women.

Prospective cohort studies are necessary to assess violence and mental health during pregnancy. Moreover, it would be important to evaluate the dietary pattern of pregnant women exposed to violence in view of its impact not only for the mothers but also for their offspring. A recent study showed that physical violence by an intimate partner was associated with greater adherence to a dietary pattern of lower nutritional quality among pregnant women from a Brazilian cohort^(50)^.

## Conclusion

The relationship between psychological violence in the last 12 months and overweight/obesity during pregnancy was mediated by mental health changes. Mental health changes mediated 46·1 % of the relationship between psychological violence and overweight/obesity. The findings of this original study show that nutritional status is not limited to biological factors and highlights the importance of mental and psychological factors.

It is very important to detect situations of violence and mental health changes in pregnancy because of the intense physiological changes during this period of life, which probably render the pregnant women more vulnerable to these events. Furthermore, we emphasise the need for multiple antenatal screenings to detect DV and mental health changes at an early stage of pregnancy in order to develop safe and appropriate interventions that will prevent complications not only for the mother but also for their offspring. Finally, cohort studies assessing violence and mental health status of women throughout pregnancy are needed to evaluate the impact of these events during the different trimesters of pregnancy on maternal and child health.
